# Update on completed and ongoing trials investigating TORS in head and neck oncology

**DOI:** 10.3389/fonc.2026.1863731

**Published:** 2026-08-18

**Authors:** Annelien Boonen, Fien Verstappen, Sandra Nuyts, Paul M. Clement, Jeroen Meulemans, Davide Di Santo, Robin Willaert, Auke van Mierlo, Kristof Menten, Christophe Vanclooster, Peter Neyt, Vincent Vander Poorten

**Affiliations:** 1 University Hospitals Leuven, UZ Leuven, Leuven, Belgium; 2 Katholieke Universiteit Leuven, Leuven, Belgium; 3 Ziekenhuis Oost-Limburg, Genk, Belgium; 4 AZ Sint-Lucas & Volkskliniek, Ghent, Belgium

**Keywords:** circulating cell-free HPV DNA (cfHPVDNA), clinical trials, head and neck cancer, HPV-associated cancer, oropharyngeal squamous cell carcinoma (OPSCC), risk stratification, transoral robotic surgery (TORS), treatment de-escalation

## Abstract

**Background:**

Human papillomavirus (HPV) has substantially altered the epidemiology and prognosis of oropharyngeal squamous cell carcinomas (OPSCC), with an increasing incidence in younger patients and markedly improved survival outcomes. This shift presents an increasing clinical challenge: how to safely reduce treatment intensity, while maintaining cure and survival rates and minimizing therapy-related toxicity. Transoral robotic surgery (TORS) has emerged as a pivotal strategy in this context, with multiple clinical trials demonstrating promising outcomes. This review aims to summarize registered clinical trials evaluating TORS in OPSCC, identify existing gaps in the literature, and highlight directions for future research.

**Methods:**

A systematic search of *ClinicalTrials.gov* was conducted between May 2024 and January 2026 using the search terms “TORS” and “transoral robotic surgery.” The search included completed and analyzed studies, as well as completed but unanalyzed, ongoing or planned studies. Relevant publications were retrieved from databases including *ClinicalTrials.gov*, PubMed and The Cochrane Library. As this study was based on publicly available literature and trial registries, ethical committee approval was not required.

**Results:**

A total of 51 clinical trials were identified. We retrieved a total of 23 publications and 3 abstracts. Across these studies, TORS demonstrated oncologic outcomes comparable to radiotherapy in early-stage OPSCC, with distinct but generally acceptable toxicity profiles. Several trials supported the feasibility of treatment de-escalation, including reduced-dose or omission of adjuvant radiotherapy in carefully selected HPV-positive patients, without compromising survival outcomes. Emerging evidence from biomarker-driven studies suggests that circulating cell-free HPV DNA (cfHPVDNA) may enable improved risk stratification and guide personalized de-intensification strategies. In addition, optimization studies indicate that advances in perioperative management and robotic technology may further improve functional outcomes, although high-quality evidence remains limited. Five key areas of research were identified, namely the optimization strategies, TORS as an alternative strategy, TORS for de-escalation, biomarker-driven de-escalation, and induction therapy.

**Conclusion:**

Transoral robotic surgery demonstrates favorable oncological and functional outcomes, supporting its role as a de-intensification strategy in carefully selected patients with OPSCC. The integration of robust risk stratification methods, including biomarkers such as circulating cell-free HPV DNA (cfHPVDNA), holds promise for optimizing patient selection and tailoring treatment intensity. Continued advancements in robotic technology, along with the development of refined perioperative pain management strategies, may further enhance surgical precision and postoperative recovery.

## Introduction

Head and neck squamous cell carcinoma (HNSCC) diagnoses are rising at an alarming rate, with an incidence over 800–000 and over 400–000 deaths annually. It has become the seventh most common cancer worldwide, and global incidence is projected to increase by more than 40% by 2040 according to the Global Cancer Observatory (1). A significant driver of this trend is the rise of HPV-associated oropharyngeal squamous cell carcinoma (OPSCC). Oncogenic HPV types - most notably HPV-16 and HPV-18 - have become the primary cause of OPSCC in high-income countries, predominantly affecting younger white males of middle socioeconomic status. In low-income countries, tobacco and alcohol remain the principal etiologic factors, typically affecting older individuals and of lower socioeconomic background (2). Despite its increasing burden, HPV-positive OPSCC offers a more favorable prognosis than HPV-negative disease, demonstrating improved overall survival (OS), progression-free survival (PFS) and recurrence rates (RR). This prognostic advantage is thought to reflect the greater sensitivity of HPV-associated tumors to current standard treatments, rather than an inherently less aggressive biological behavior (3, 4). Importantly, not all de-escalation strategies have proven successful: trials replacing cisplatin with cetuximab failed to demonstrate non-inferiority, even in patients classified as low risk, underscoring the need for careful patient selection and validated de-escalation approaches (5). The younger age of many affected patients further underscores the growing need to reassess current treatment strategies and to reduce therapy-related morbidity without compromising oncologic efficacy (3, 4).

In this respect, treatment of OPSCC has evolved from traditional open surgical approaches towards radiotherapy (RT) and chemoradiotherapy (CRT), which remain standard of care but can be associated with significant toxicity including xerostomia, osteoradionecrosis, mucositis, sepsis and long-term swallowing dysfunction. However, advances in radiation techniques, such as intensity-modulated radiotherapy (IMRT), volumetric modulated arc therapy (VMAT), and intensity-modulated proton therapy (IMPT), have substantially improved dose conformity and have been shown to reduce the risk of treatment-related toxicity. Minimally invasive techniques, such as transoral robotic surgery (TORS) and transoral microscopic laser surgery (TML), emerged in the early 2000s as alternative strategies. TORS, first described by O’Malley et al. in 2006 (6), provides superior visualization and access within the anatomically confined oropharynx, reducing the technical limitations and complications associated with open surgery. Following the Food and Drug Administration’s (FDA) approval in 2009 for T1-T2 cancers, the Da Vinci robotic system became widely adopted as a key treatment option for early stage oropharyngeal cancer (7, 8).

**Figure 1:**
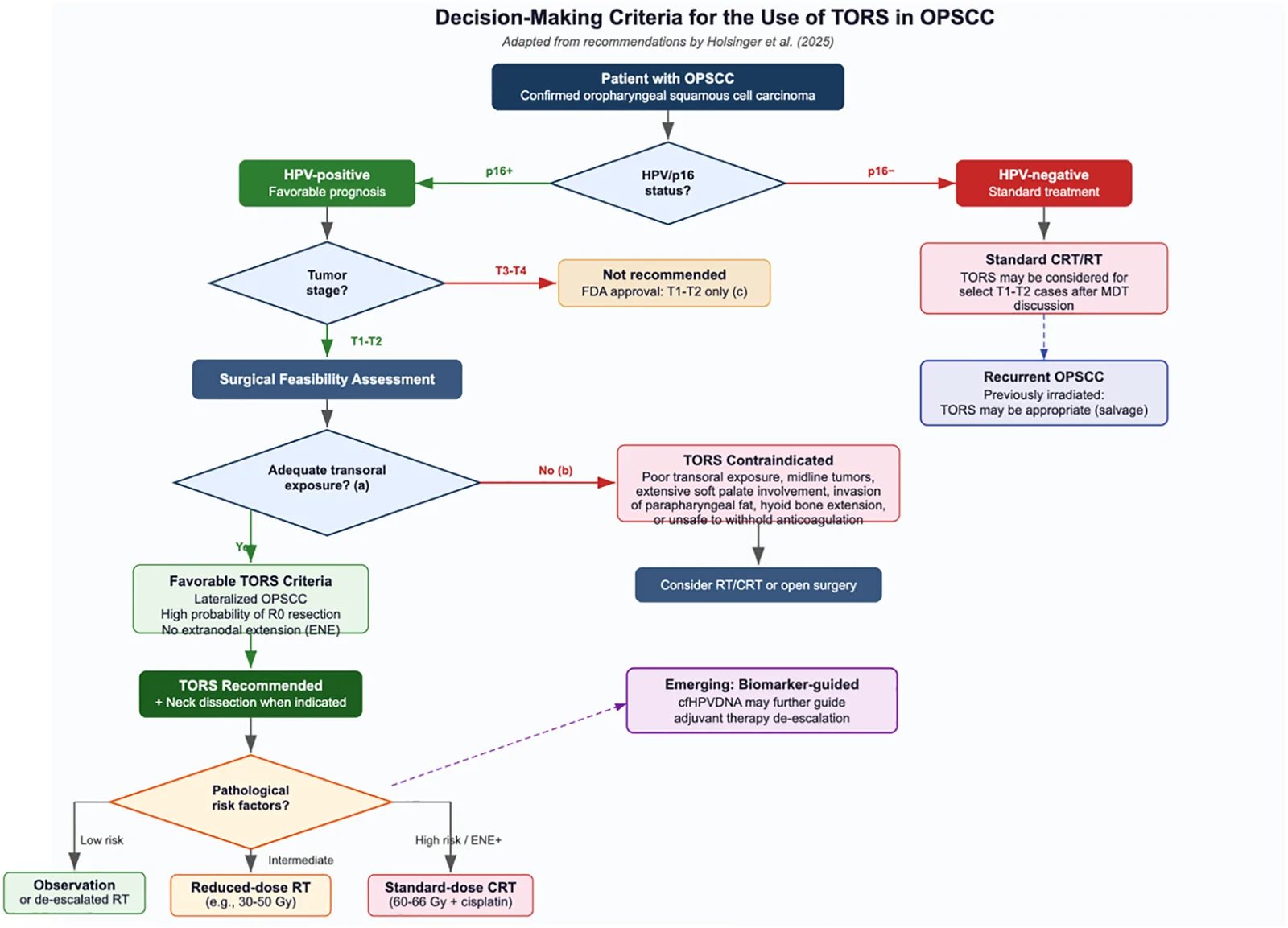
Decision making criteria for the use of TORS. **(a)** Adequate transoral exposure, lateralized OPSCC, high probability of R0 resection based on multimodal preoperative assessment. **(b)** Poor transoral exposure, midline tumors, extensive soft palate involvement, parapharyngeal fat invasion, hyoid bone extension, extrinsic tongue musculature involvement, or inability to safely withhold anticoagulation. TORS is currently approved by the FDA for T1-T2 tumors only. Adapted from recommendations in: Holsinger FC et al. J Clin Oncol. 2025;43 (9):1369-1392. doi:10.1200/JCO-24-02755 (8). CRT, chemoradiotherapy; cfHPVDNA, circulating cell-free HPV DNA; ENE, extranodal extension; MDT, multidisciplinary team; OPSCC, oropharyngeal squamous cell carcinoma; RT, radiotherapy; TORS, transoral robotic surgery.

Given the shift in OPSCC epidemiology and the excellent prognosis associated with p16-positive disease, there is a growing emphasis on developing less intensive therapies that maintain equivalent survival rates while reducing toxicity and functional impairment. As p16 immunohistochemistry is widely accepted as a surrogate marker for transcriptionally active HPV, its use has become integral to contemporary risk stratification and the design of de-escalation trials. TORS has shown promising results in these de-escalation strategies and has become a focus of numerous clinical trials. The recently published ASCO guideline (2025) provides specific recommendations regarding the use of TORS in clinical practice (8). Current decision-making criteria for selecting TORS are summarized in **Figure 1**. At present, TORS is recommended for carefully selected patients with low-stage OPSCC, depending on adequate transoral exposure and the likelihood of achieving complete resection, and it may also be appropriate for recurrent tumors.

To date, several published and ongoing studies explore expanding the role of TORS in oropharyngeal cancer. Recently, interest has grown in circulating cell-free HPV DNA (cfHPVDNA) as a post-treatment surveillance biomarker that may enable earlier detection of residual disease or recurrence following TORS, thereby supporting more personalized, risk-adapted decisions regarding adjuvant therapy intensity (10, 11). Additionally, research continues to investigate robotic mechanics and the application of TORS in higher-stage OPSCC.

This review aims to provide a structured overview of registered clinical trials investigating TORS in OPSCC, to identify gaps in the current evidence base, and to highlight key directions for future research. Although the primary focus is OPSCC, trials investigating TORS in supraglottic squamous cell carcinoma (SGSCC) and carcinoma of unknown primary (CUP) were also included given the anatomical and oncological overlap with OPSCC. Clinical trials were categorized into five key areas: optimization strategies, TORS as an alternative treatment, TORS for de-escalation, biomarker-guided de-escalation and induction therapy.

## Methods

### Search strategy

To address our research question, we conducted a literature search in *ClinicalTrials.gov* between May 2024 and January 2026 using the search terms “*TORS*” or “*transoral robotic surgery*”. No additional filters were applied, ensuring the inclusion of completed and analyzed studies, completed but unanalyzed studies, as well as ongoing and planned clinical trials. A total of 132 records were retrieved, which were subsequently deduplicated and screened based on title and study characteristics (AB, FV and VV).

Inclusion criteria were:

- use of TORS as therapeutic modality and
- a study focus on head and neck malignancies, including OPSCC, supraglottic squamous cell carcinoma (SGSCC), and carcinoma of unknown primary (CUP).

Exclusion criteria were:

- TORS for obstructive sleep apnea,
- TORS for pituitary gland surgery,
- TORS for thyroid surgery,
- study status unknown or terminated, and
- study status: completed, but low accrual.

Relevant publications and additional study information were subsequently retrieved from databases including PubMed, The Cochrane Library, conference proceedings (ASCO; ESMO) and *ClinicalTrials.gov*. Since this was a literature review, approval from an ethical committee was not required.

## Results

### Screening process

This review was conducted in accordance with scoping review methodology. In the initial phase, we screened clinical trials and associated articles based on subject, study design, title and abstract. We then performed a comprehensive review of the full texts of the eligible records. This process resulted in a final database of 51 studies. We retrieved 23 publications and 3 abstracts.

**Figure 2:**
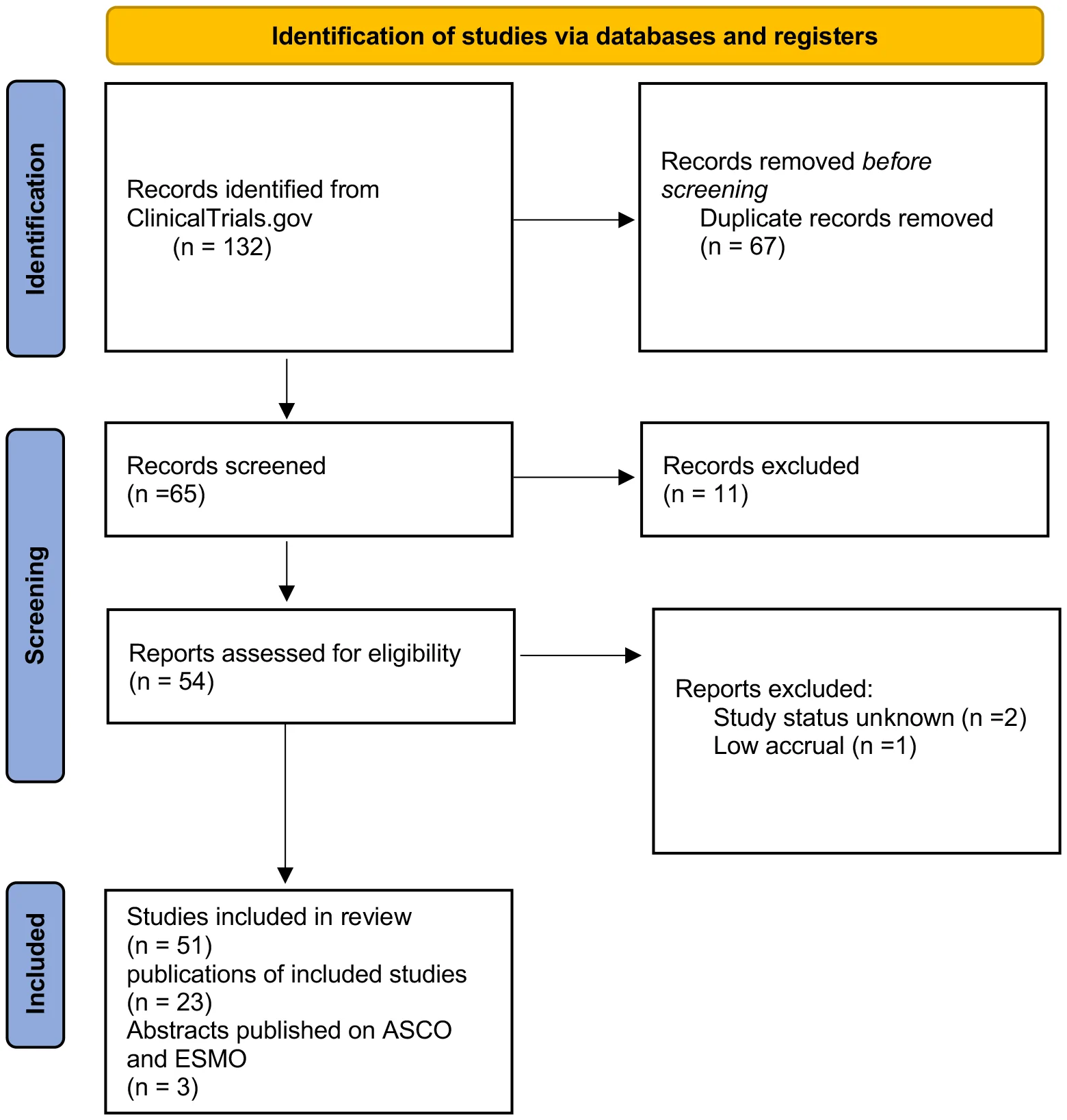
PRISMA flow diagram of study selection process.

A PRISMA flow diagram summarizes the study selection process at each stage (**Figure 2**).

A total of 51 clinical trials were included in this review. All trials were categorized according to their primary research focus and are summarized in **Table 1** through 5. The tables provide an overview of published clinical trials, including study identification number, first author, year of publication, study design, sample size, intervention and key outcomes. Ongoing and unpublished trials are presented with details on study identification number, recruitment status, anticipated completion date, estimated sample size, intervention, and primary outcome measures.

**Table 1:** clinical trials evaluating the optimization of TORS in OPSCC.

Study number, first author or PI, date of publication	Study status	Study type (sample size)	Patient inclusion	Intervention	Primary outcome	Key findings
NCT01748942 Clayburgh D. Nov 2017 (8)	Completed– paper published	Randomized Pilot Study (n= 76)	OPSCC eligible for curative TORS	IO dexamethasone (10 mg IV) + TORS * + dexamethasone (8mg oral/parenteral) 3x/d for 4 days or until discharge vs IO dexamethasone (10 mg IV) + TORS * + placebo (oral/parenteral) 3x/d for 4 days or until discharge	VAS	There was no significant difference in VAS scores on POD 1, 2, and 7 through 21, but a significant difference was observed on POD 3. Opioid use was similar in both groups. A decrease in hospital stay by a median of 1 day was noted, along with improved food tolerability.
NCT04189107 Larsen M.H.H. Oct 2023 (10)	Completed– paper published	Phase 3 (n= 18)	OSAS or SCCUP	IO dexamethasone (24 mg IV) + TORS ** + dexamethasone (12 mg IV) on POD2 and POD4 vs IO dexamethasone (8 mg IV) + TORS** + placebo (IV) on POD2 and POD4	VAS	VAS scores, opioid use and food tolerability showed no significance in treatment groups.
NCT05862792 PI: Haugen T. Est. 01/2026	Recruiting	Observational Study (n= est. 80)	OPSCC	TORS + Liposomal Bupivacaine (injection into surgical wound) vs TORS	VAS	N/A
NCT03010813 NCT03049280 (11) Holsinger F.C. Nov 2019	Completed – paper published	Prospective Interventional Study (n= 47)	T1-T2 benign or malignant tumor of oropharynx	SP TORS *	Conversion rate, perioperative complications	Single port TORS is a safe and feasible device, in terms of device related serious adverse events and conversion rates.
NCT02792322 PI: Van Abel, K.	Completed 12-2024 – analysis and results pending	Interventional Pilot Study (n= 32)	TORS for benign/malign head and neck disease	TORS (Da Vinci) in seated position	Completion Rate	N/A
NCT01473784 PI: Ozer, E.Est. 09/2025	Recruiting	Interventional Pilot Study (n= est. 600)	Benign and/or malign disease of the oral cavity/laryngopharynx	TORS: Da Vinci	Feasibility	N/A
NCT06112535 PI: Fleming, J.	Completed 04-2025 - analysis and results pending	Prospective Interventional Study (n= est. 60)	pT1-3, N0–1 OPSCC (AJCC 8th ed.)	TORS: Versius Surgical System	Adverse events	N/A
NCT02517125 Contact person- Gustave Roussy, Cancer Campus, Grand Paris	Completed 04-2025 - analysis and results pending	Interventional Study (n= 100)	Head & neck cancer with transoral exposition	TORS: Da Vinci Xi	Achievement rate of transoral robotic surgery	N/A

*Transoral robotic surgery (TORS) may be complemented by adjuvant neck dissection when clinically indicated. **Lingual tonsillectomy performed by TORS.

PI, principal investigator; n, number; IO, intraoperative; IV, intravenous; d, day; VAS, Visual Analogue Scale; POD, postoperative day; OSAS, obstructive sleep apnea syndrome; SCCUP, squamous cell carcinoma of unknown primary; est, estimated; SP, Single port; N/A, not applicable; AJCC, American Joint Committee on Cancer; ed, edition;

### Optimization strategies

Despite its minimally invasive nature, TORS is associated with procedure-related morbidity, including postoperative hemorrhage, dysphagia, and postoperative pain. The latter two may lead to reduced oral intake, dehydration, aspiration, pneumonia, and prolonged hospitalization (4, 9). Consequently, several clinical trials have investigated perioperative strategies aimed at reducing postoperative complications and improving functional recovery. An overview of trials focusing on optimization strategies is provided in **Table 1**.

Two randomized studies assessed the role of perioperative corticosteroids in postoperative pain management following TORS. Clayburgh et al. (2017) investigated the effect of an extended course of dexamethasone on postoperative pain in 76 OPSCC patients undergoing TORS. Patients received either perioperative dexamethasone administered every eight hours for four days or placebo. No significant differences in Visual Analog Scale (VAS) scores on postoperative day (POD) 1, 2 or between POD 7 and POD 21 were observed. However, the steroid group showed a notable improvement in pain-level on POD 3, a steady net-improvement over 3 days, improved food tolerability (measured by Performance Status Scale (PSS)) and a shorter median hospital stay by one day. Total opioid consumption did not differ between groups (12).

Larsen et al. (2023) performed a randomized double-blinded trial in 18 patients undergoing TORS for lingual tonsillectomy, comparing high-dose and low-dose dexamethasone group. The high-dose group received 24 mg of dexamethasone perioperatively, followed by 12mg on POD 2 and 4, while the low-dose group received 8 mg of dexamethasone perioperatively, followed by a placebo. Outcomes, including VAS pain scores at rest and during swallowing, opioid use, length of hospitalization and food tolerability, showed no significant differences between treatment arms. Larsen’s findings showed no difference in pain levels on POD 3 or during the first week and no difference in food tolerance, unlike Clayburgh et al. (13).

An ongoing observational study (NCT05862792) is currently recruiting patients to evaluate the effect of liposomal bupivacaine injection into the surgical bed during TORS. Primary outcomes include postoperative pain and dysphagia, assessed using VAS scores and analgesic requirements, as well as functional endoscopic swallowing assessment and patient-reported outcome measures. Results are anticipated in the beginning of 2026.

### Robotic mechanics

Advances in robotic mechanics represent another avenue for optimizing TORS outcomes.

Since the FDA approval of the Da Vinci robotic system (Intuitive Surgical, Inc., Sunnyvale, CA) for transoral surgery in 2009, subsequent generations of robotic surgical systems aim to overcome technical difficulties regarding visualization and instrument maneuverability within the confined anatomy of the oropharynx.

Holsinger et al. (14) evaluated the safety and feasibility of the next-generation single-port (SP) robotic system across two prospective interventional trials (NCT03010813 and NCT03049280). This SP system allows surgeons to insert 3 fully articulating 6mm instruments and a 1.2-cm stereo-endoscope camera through a single port. A total of 47 patients with benign or malignant T1–T2 oropharyngeal tumors underwent SP TORS. No intraoperative complications, device-related serious adverse events, or conversions to alternative surgical approaches were reported. The positive margin rate was 3%, mean estimated blood loss was 15.4 mL, and no blood transfusions were required. These results indicated the SP system as safe and feasible device for oropharyngeal tumors (13).

Additional technical studies are ongoing or awaiting data maturation and analysis. A completed pilot study (NCT02792322) evaluated the feasibility of performing TORS with patients in a seated position facilitating better visualization, with completion rate as the primary endpoint. The study completed accrual in December 2024; results have not yet been published. In addition, a large prospective study (NCT01473784), led by investigators at the Ohio State University and initiated in November 2011, is currently enrolling approximately 600 patients to assess feasibility, surgical utility, and learning curves associated with TORS for benign and malignant oral and laryngopharyngeal lesions. Results are expected at the end of 2026. Furthermore, feasibility studies evaluating alternative robotic platforms, including the Versius robotic (CMR surgical^®^) system (NCT06112535), and the da Vinci Xi system (NCT02517125), have completed accrual and are awaiting analysis.

### TORS as an alternative treatment strategy

In early-stage OPSCC (T1-T2), standard treatment options include RT with or without chemotherapy, or primary surgery using TORS combined with neck dissection and, when indicated, adjuvant (chemo)RT. Each approach has specific toxicity profiles. RT and CRT can cause, among others, xerostomia, dysphagia, osteoradionecrosis, trismus, mucositis and sepsis. TORS presents risks like dysphagia, bleeding, pain, and nerve damage. Comparing functional and oncologic outcomes aids shared decision making regarding the choice of treatment. **Table 2** summarizes clinical trials on TORS as alternative strategy.

**Table 2:** clinical trials evaluating TORS as alternative treatment strategy.

Study number, first author or PI, date of publication	Study status	Study type (sample size)	Patient inclusion	Intervention	Primary outcome	Key findings
NCT01590355 (ORATOR) Nichols A.C. Aug 2019 (9, 12, 13)	Completed	Phase 2 (n= 68)	T1-T2, N0-N2 (≤4 cm) OPSCC (AJCC 7th ed.)	70Gy RT +/- CT (if N1, N2) vs TORS* +/- adjuvant RT/CRT	QOL	There were no differences in oncological outcomes between both groups, however p16 positivity was associated with improved OS and PFS compared to p16-negativity. Swallowing function-related quality of life (QOL), as measured by the MDADI score, was superior in the RT group at 1 year, although not clinically significant. Differences visibly converged over time (up till 5 years). The toxicity profiles of both treatments differed, with more neutropenia and hearing loss in the RT group, and more dysphagia and pain in the TORS group.
**NCT03210103 (ORATOR2) Palma D.A. Apr 2022**	Completed (stopped early)	Phase 2 RCT (n = 61)	T1–T2, N0–N2, p16+ OPSCC (AJCC 8th ed.)	60 Gy RT ± concurrent weekly cisplatin (node-positive) vs TOS (TORS or TLM)* + ND + risk-adapted adjuvant RT (50 Gy/25 fx, or 60 Gy/30 fx if positive margins or ENE)	OS vs historical control	Trial halted due to excess toxicity in TOS arm. Two treatment-related deaths (oropharyngeal haemorrhage day 4; cervical vertebral osteomyelitis day 110). All oncologic events in TOS arm; OS/PFS data immature. Grade 2–5 toxicity comparable (67% RT vs 71% TOS). MDADI at 1 year similar (85.7 vs 84.7; P = .85). Surgery-first approach not recommended in this population pending mature survival data.
NCT03418909 Scott S.I. Aug 2023 (13)	Completed	Prospective Cohort study (n= 44)	T1-2, N0-1, M0 OPSCC (AJCC 7th Ed)	TORS* vs RT	Salivatory function	Both arms noted a decrease in objective swallowing function from baseline to 1-year and improvement of swallowing function up to 3 years. TORS demonstrated significant improvements in swallowing safety and efficiency. In the radiation group only significant improved efficiency was seen, although MDADI scores deteriorated from 1 to 3 y follow up.
NCT03357107 Doazan M. Sep 2018 (15)	Completed	Observational study (n= 122)	T1-T3, M0 SGSCC	TORS +/- adjuvant (60–66 Gy RT +/- chemotherapy)**	Local control	Oncological outcomes, including OS, PFS, LRCR and LCR showed comparable oncological outcomes to standard of care treatment options. Adjuvant therapy was obligatory in 51.6% of the cases, of which 25% was due to close or positive margins.
NCT01059357 Hughes R.T (14). Aug 2023	Completed	Interventional Pilot Study (n= 167)	T1-T2, N0-N2, stage I-II HPV+/p16+ OPSCC (AJCC 8th Ed)	TORS* +/- adjuvant RT/CRT vs RT (70 Gy) +/- CT	Successful completion	For early stages of HPV-positive OPSCC, there were no significant differences in OS, PFS, and FOIS. This remained consistent after adjusting for stage, age, and comorbidity. The radiotherapy group showed a higher rate of GT dependence. Clinical nodal stage was significantly associated with a greater change in FOIS
NCT02663583 Gunn G.B. 2020 (ABSTRACT ASCO) (16)	Completed	Observational Study (n= 44)	Stage I/II/III OPSCC (AJCC 7th Ed)	TORS vs IMPT	MDADI, Activity monitoring	Both arms showed a significant decrease in activity at 3 months and in caloric intake at 6 months. There was no significant difference between two arms.
NCT04673929 (RECUT) Hardman J.C. Feb 2022 (17)	Completed	Observational Study (n= 278)	Recurrent HNC, previously treated with RT	TORS	DSS, DFS	TORS for recurrent head and neck cancer, previously treated by radiation, showed excellent functional and survival outcomes. Significantly associated parameters with OS were increasing age, current smoking, primary tumor classification and narrow close margins (<=1.0 mm)
NCT02984410 (EORTC 1420, Best of) Study chair: Simon, C. Est. 09/2029	Active, not recruiting	Phase 3 (n= 112)	T1-T2, N0-N1 (one single neck node ≤ 3cm), M0 OPSCC, SGSCC, HPSCC	transoral surgery (TORS/TLM/CTOS) vs IMRT	MDADI	N/A
NCT04124198 (QoLATI) PI: Rubek, N. Est. 01/2029	Active, not recruiting	Phase 3 (n= est. 138)	P16+ cT1–2 cN0–1 OPSCC (AJCC 8th ed.)	TORS* vs IMRT	MDADI	N/A
NCT05611515 (Supra-QOL) PI: Hassid, S. Est. 12/2026	Recruiting	Observational Study (n= est. 108)	cT1-T2/cN0-N1/M0 supraglottic (AJCC 8th ed.)	IMRT vs TLM vs TORS	MDADI	N/A
NCT05933889 (SCORE) PI: Paleri, V. Est. 08/2027	Recruiting	Observational Study (n= Est. 250)	P16+/- OPSCC, pT1-T2	TORS +/- RT + Molecular analysis + Radiomic analysis	LRS	N/A
NCT05341479 Study chair: Tao, L. Est. 07/2025	Recruiting	Observational Study (n= Est. 200)	T1-T4 OPSCC	T1-2, N0: TORS/TLM/CTOS/open surgery * +/- RT vs RT T1-2,N1-3/T3-4,N0-3: CRT vs TORS/TLM/CTOS/open surgery * +/- RT vs neoadjuvant treatment + CRT/surgery +/- adjuvant therapy	OS	N/A
NCT05144100 (SCOPE)PI: Deepal, N. Est. 06/2031	Not yet recruiting	Phase 2 Phase 3 (n= est. 498)	HPV negative stage III-IV OPSCC	surgery (TORS/TLM/open/endoscopic) * vs CRT (40 mg weekly + 66–70 Gy)	OS, EFS	N/A
NCT04952922 (FUNQOLR) Royal Marsden NHS May 2024	Completed	Observational Study (n= 25)	Recurrent OPSCC	Open surgery, TORS, palliative chemotherapy, immunotherapy	MDADI	N/A
NCT04638465 Nebraska Methodist Hospital Est. 01/2030	Active Not recruiting	Observational Study (n= est. 150)	HPV+ SCC (AJCC 8th ed.)	TORS vs TORS + cisplatin vs CRT (cisplatin 40 mg/m2 + 60 Gy) vs CRT (cisplatin 40 mg/m2 + 70 Gy)	OS, DFS	N/A
NCT01819480 PI: Fedder, K.L. Est. 12/2025	Active not recruiting	Interventional Study (n= est. 110)	OPSCC/SGSCC, M0	TORS: Da Vinci	PFS	N/A
**NCT04671940** (RECUT PLUS) PI: Paleri, V. Est. 10/2026	Recruiting	Observational Study (n = est. 20)@	TORS for recurrent HNC previously treated with RT	DNA analysis + TORS + DNA analysis	Identification of resistant subclones	N/A

*Transoral robotic surgery (TORS) may be complemented by adjuvant neck dissection when clinically indicated.

**if more than 1 lymph node, positive margins, extracapsular nodal spread, perineural or lymphovascular invasion

PI, principal investigator; n, number; AJCC, American Joint Committee on Cancer; ed, edition; QOL, quality of life; OS, overall survival; PFS, progression free survival; MDADI, MD Anderson Dysphagia Inventory; SGSCC, supraglottic squamous cell carcinoma; LRCR, locoregional control rate; LCR, local control rate; FOIS, Functional Oral Intake Scale; GT, gastrostomy tube; IMP, Intensity Modulated Proton Therapy; HNC, head and neck cancer; DSS, disease specific survival; DFS, disease free survival; HPSCC, Hypopharyngeal Squamous Cell Carcinoma; TLM, transoral Laser Microsurgery; CTOS, Conventional Transoral Surgery; IMRT, Intensity Modulated Radiation Therapy; N/A, not applicable; LRS, Locoregional survival; EFS, event free survival; OPSCC, oropharyngeal cancer; SCC, squamous cell carcinoma;

The ORATOR (**O**ropharyngeal cancer **R**adi**AT**i**O**n vs **R**obotic surgery) trial (NCT01590355) was the first randomized study comparing primary RT with primary TORS plus neck dissection in patients with T1–T2 OPSCC. Sixty-eight patients were randomized to receive either 70 Gy RT or TORS with neck dissection, followed by adjuvant therapy when indicated. At one year, MD Anderson Dysphagia Inventory (MDADI) scores were higher in the RT group, although the predefined threshold for clinical significance was not reached ((86.9 vs 80.1; p*=*0.042). Differences diminished over longer follow-up (2 years: 86 vs 85; p=0.74), and five-year results demonstrated comparable oncologic outcomes between treatment arms (14–16). Notably, the trial enrolled patients with up to N2b disease according to the 7th edition TNM classification, a subgroup for which adjuvant RT would have been indicated regardless of the surgical approach. This inclusion of higher-risk nodal disease may limit the interpretation of results in the context of de-escalation, as a substantial proportion of TORS patients would inevitably require multimodal therapy (17).

The ORATOR2 trial (NCT03210103) was an international, multicenter phase II randomized clinical trial conducted at nine academic cancer centers in Canada and Australia, randomizing 61 patients with HPV-positive OPSCC (T1–T2, N0–N2, p16-positive, AJCC 8th edition) in a 1:1 ratio to primary CRT (60 Gy ± concurrent weekly cisplatin in node-positive patients) or TOS plus neck dissection followed by risk-adapted adjuvant RT based on pathological findings. Notably, 78% of patients in the surgical arm ultimately required adjuvant radiotherapy. Accrual was halted in November 2020 due to excessive toxic effects in the TOS arm, including two treatment-related deaths from oropharyngeal hemorrhage and cervical vertebral osteomyelitis. At a median follow-up of 17 months, all oncologic events occurred exclusively in the surgical arm, leaving OS and PFS data immature. Grade 2–5 toxicity was comparable between arms (67% vs 71%), and one-year MDADI scores were similar (85.7 vs 84.7; P = .85). These findings indicate that a surgery-first approach carries an unacceptable risk of grade 5 toxic effects in this population, and long-term follow-up data are awaited (18).

A prospective cohort study (NCT03418909 - 2023) objectively evaluated swallowing function in 44 patients undergoing TORS or non-surgical treatment. Both groups experienced similar declines in swallowing function from baseline to one year, followed by improvement by three years. Swallowing efficiency and safety improved significantly in the TORS group, whereas the RT group demonstrated improvement in efficiency only. Contrary to the ORATOR trial, MDADI scores in the TORS arm improved at three years but deteriorated in the RT group (19).

Hughes et al. reported outcomes from an interventional pilot study (NCT01059357 - 2023) including patients with early-stage HPV-positive OPSCC. No significant differences were observed between TORS-based and RT-based treatment strategies in overall survival, progression-free survival, or functional oral intake scale scores at one year. N2 nodal status at diagnosis was an independent risk factor for poorer swallowing function (20).

NCT02663583 focused on longitudinal activity monitoring after Intensity Modulated Proton Therapy (IMPT) and TORS. Early results published as an abstract in ASCO 2020, showed decreased physical activity at three months and caloric intake at six months, with no statistically significant differences between treatment groups. Further results are pending (21).

Beyond OPSCC, an observational study (NCT03357107, 2018) evaluated TORS in 122 patients with T1-T3 supraglottic squamous cell carcinoma, reporting high rates of local and locoregional control at two and five years (local control rates of respectively 94.3% and 90.2%, and locoregional control rates of respectively 91.8% and 87.7%). Adjuvant therapy was administered to 63 patients (51.6%), primarily due to positive surgical margins (13.9%) or nodal invasion (74.6%). These findings support TORS as a valuable alternative for early-stage supraglottic tumors, expanding its applicability (22).

The RECUT study (NCT04673929 - 2022) followed 278 patients with recurrent head and neck cancer (HNC), previously treated with RT, all of whom underwent TORS. At two and five years, locoregional control was 69.0% and 62.2%, OS was 71.8% and 49.8%, disease free survival (DFS) was 47.2% and 35.7%, and disease specific survival (DSS) was 78.7% and 59.1%. Margins ≤1.0 mm had lower LRCR (54.2%) than margins >1.0 mm (80.9%). Tracheostomy and gastrostomy rates dropped significantly from perioperative levels (42.9%) to one-year rates (7% and 32.4%) (23).

The RECUT PLUS study (NCT04671940) investigates the impact of prior radiotherapy on recurrent, residual, or second primary HNSCC. This study analyzes DNA characteristics of tumor tissue and subclonal populations before and after radiotherapy to elucidate molecular mechanisms of radiation resistance and refine risk stratification approaches. Study completion is anticipated in October 2026.

The SCORE trial (NCT05933889) has just started recruiting data on 250 patients with pT1-T2 OPSCC treated with TORS, evaluating survival outcomes, safe margin cutoffs, complication rates and mortality. Another key aspect is incorporating molecular analysis to distinguish recurrent from non-recurrent OPSCC and evaluate circulating tumor DNA before and after TORS. Completion is expected in August 2027.

NCT01819480 is investigating the safety and efficacy of TORS in 110 patients with OPSCC/SGSCC, with PFS at three years as the primary endpoint. Results are pending.

Several ongoing trials further evaluate transoral surgery in comparison with intensity-modulated RT (IMRT), with a primary focus on functional outcomes. The BEST-OF trial (NCT02984410), has enrolled 112 patients diagnosed with low-stage OPSCC, SGSCC, or HPSCC. Participants were stratified into either an IMRT or a surgery arm. The surgery group includes TORS, transoral laser microsurgery, or conventional transoral surgery. The primary outcome is swallowing function, assessed by MDADI scores up to one year. The Supra-QOL (**Supra**glottic tumors – **Q**uality **O**f **L**ife) study (NCT05611515) specifically focuses on supraglottic tumors and assigns patients to one of three treatment strategies: TORS, transoral laser microsurgery, or IMRT. Swallowing-related quality of life, measured by MDADI, is the primary outcome, with follow-up planned through two years. The QoLATI (**Q**uality **o**f **L**ife after **A**lternative **T**reatment **I**nterventions) study (NCT04124198) is a randomized study designed to assess functional and oncologic outcomes in patients with T1–T2 p16-positive OPSCC undergoing TORS with neck dissection versus IMRT, using a 2:1 allocation. A total of 138 patients have been enrolled and are currently followed for one year. Primary outcome measures are summarized in **Table 2**. Anticipated completion dates are September 2029 for BEST-OF, December 2026 for Supra-QOL, and January 2029 for QoLATI.

Additional large observational studies are ongoing in both HPV-positive and HPV-negative populations. A prospective Chinese observational clinical trial (NCT05341479) aims to enroll 200 early- and late-stage OPSCC patients and evaluates OS based on clinical stage and HPV expression. Patients are managed with RT, surgery, or neoadjuvant therapy followed by RT or surgery. Results were anticipated by October 2025, but since registration on *ClinicalTrials.gov*, no updates have been posted. The Indian SCOPE trial (NCT05144100), registered in December 2021, plans to recruit 498 stage III-IV HPV-negative OPSCC patients, to compare surgical and CRT- based strategies, with outcomes expected in June 2031. Recruitment has not yet commenced, and completion is anticipated in June 2031. Additionally, trial NCT04638465 is an observational study aiming to classify 150 patients with HPV-positive squamous cell carcinoma (SCC) (using the AJCC 8^th^ edition staging system) to evaluate the effects of de-escalated treatments, with completion anticipated in 2030.

Finally, the FUNQOLR (FUNctional QOL in Recurrence) trial (NCT04952922) evaluated swallowing-related quality of life in 25 patients receiving treatment for recurrent oropharyngeal cancer; the study included both curative and palliative intent treatments. Given the mixed intent population, results should be interpreted with caution in a curative context; results are pending.

### TORS as de-escalation strategy

#### Reduction of adjuvant radiotherapy

Given the favorable prognosis of HPV-positive oropharyngeal squamous cell carcinoma (OPSCC), multiple clinical trials have investigated the role of transoral robotic surgery (TORS) as a platform for reducing the intensity of adjuvant therapy while maintaining oncologic outcomes. Clinical trials evaluating adjuvant treatment de-escalation are summarized in **Table 3**.

**Table 3:** clinical trials evaluating TORS as part of reduction in adjuvant therapy.

Study number, first author of PI, date of publication	Study status	Study type (sample size)	Patient inclusion	Intervention	Adjuvant RT de-escalation strategy	Primary outcome	Key findings
NCT01932697 (MC1273) Jun 2019 Ma D.J (19).	Completed	Phase 2 (n= 80)	P16+ stage III-IV OPSCC and surgery with negative margins (AJCC 7th edition)	TORS + RT (30 Gy) + docetaxel (15mg/m2) +/- 36 Gy boost to nodal levels with extranodal extension	Reduced-dose RT (30 Gy adjuvant RT ± nodal boost to 36 Gy in case of extranodal extension)	LRCR	Excellent survival and functional outcomes were achieved by aggressive reduction in adjuvant dose radiotherapy after surgery. Short and long-term toxicity were significantly favorable compared to historical data.
NCT02159703 (AVOID) Swisher-McClure S Nov 2019 (22)	Completed	Phase 2 (n= 60)	pT1-pT2 N1–3 HPV+ OPSCC	TORS* + RT ipsilateral neck (60–66 Gy) + RT contralateral neck (54 Gy) +/- CT	Selective omission of RT to the primary tumor bed	LCR	Omission of radiation on resected primary tumor site after TORS showed excellent survival rates, while suggesting a significantly reduced overall radiation dose. Toxicity rates indicated favorable results.
NCT03281499 (FIND) De Almeida J.R. Apr 2024 (23)	Completed	Phase 2 (n= 22)	T0,N1-3,M0 p16+ CUP	Diagnostic/therapeutic TORS + Negative margins/pT0: PSRT Unilateral lymphadenopathy with lateralized primary tumor or pT0: UNRT	Pharyngeal-sparing RT (omission of RT to primary site in pT0/negative margins	rate of out-of-field failures	PSRT reported significantly improved swallowing function compared to non-PSRT, revealed by MDADI scores. TORS had an overall detection rate of 77%.
NCT02072148 (SIRS) Miles B.A. Jun 2021 (20)	Completed	Phase 2 (n= 54)	P16+ T1N0-2B, T2N0-2B OPSCC (AJCC 7th edition)	TORS* + surveillance/RT vs reduced RT (50 gy) vs reduced CRT (56 Gy + cisplatin)	Risk-adapted RT (no RT vs reduced-dose RT vs standard-dose CRT)	DSS, PFS, LRCR	TORS associated by risk adapted adjuvant therapy was associated with excellent survival, functional outcomes and reduced toxicity profiles. TORS followed by CRT had the slowest recovery in swallowing function.
NCT01898494 *(ECOG 3311)* PI: Ferris, R.L. Sep 2021 (21)	Active Not Recruiting	Phase 2 (n= 519)	P16+, cT1-2, N1-2b OPSCC	TOS* + low risk: observation vs intermediate risk: IMRT 50 Gy/25Fx vs Intermediate risk: IMRT 60 Gy/30 Fx vs High risk: IMRT 66 Gy/33 Fx + CDDP 40 mg/m2 weekly	Risk-stratified dose reduction (observation vs 50 Gy vs 60 Gy vs 66 Gy + cisplatin)	PFS, adverse events	Primary TOS and reduced postoperative RT result in outstanding oncologic outcome and favorable functional outcomes in intermediate-risk HPV-positive OPSCC.
NCT02908477 (DART-HPV) (24) PI: Ma, D.J.	Completed – paper published (2025)	Phase 3, open-label, randomized (n= 194)	P16+/HPV+	DART: 30–36 Gy BID/2wk + docetaxel 15 mg/m² vs SOC: 60 Gy QD/6wk + cisplatin 40 mg/m²	Reduced-dose adjuvant RT (30 Gy); ENE+ boost to 36 Gy	Grade 3 adverse events	Cumulative chronic grade ≥3 toxicity: 3% (DART) vs 11% (SOC) (p=0.042). 2-year OS: 96.1% vs 97.0%. 2-year PFS: 92.3% (met noninferiority). ENE-negative: PFS 97.7%. ENE-positive/pN2: PFS 85.2%; de-escalation not recommended for pN2 disease.
NCT02215265 PATHOS (25) PI: Evans, M.; Jones, T. Est. 04/2028	Active not recruiting	Phase 3 (n= est. 1269)	T1-T3, N0-N1 OPSCC (AJCC 8th ed.)	TLM/TORS/EATOS/lateral oropharyngectomy + ARM A: observation ARM B1: PORT 60 Gy/30 Fx ARM B2: PORT 50 Gy/25 Fx ARM C1: PORT 60 Gy/30 Fx + cisplatin ARM C2: PORT 60 Gy/30 Fx	Risk-adapted RT de-escalation (observation vs reduced-dose RT vs standard-dose RT ± cisplatin)	MDADI	N/A
NCT06578871 (SUPERIOR trial) PI: Mendez, A.I.; Jervis-Bardy, J.	Not Yet Recruiting	Phase 2 (n= Est. 90)	P16+ SCCUP, N1 (AJCC 8th edition)	TORS + IMRT to mucosa ad risk (54Gy) and ipsilateral neck (60Gy) vs N1≤ 3cm: no IMRT vs N1> 3cm or N>1: IMRT to ipsilateral neck (60 Gy)	Omission of RT to primary tumor site (no IMRT or RT limited to ipsilateral neck)	Rate of Primary Emergence of p16+ SCC	N/A
NCT06554158 PI: Ahn, P. Est. 12/2028	Recruiting	Phase 2 (n= est. 30)	P16+/HPV+ OPSCC, T1–2 N1 M0 stage I or T3 N0–1 M0 stage II (AJCC 8th ed.)	TORS + RT 50Gy to neck at risk	Target volume reduction (50 Gy to neck at risk, omission of RT to primary site)	PFS	N/A
NCT06167291 (LONE-RANGR2) PI: Rosenthal, D. Est. 12/2027	recruiting	Phase 2 (n= est. 240)	p16+/HPV+ HNSCC of the base of tongue or faucial tonsil cT1-2N0-1 (AJCC 8th ed.) No evidence of contralateral neck disease	ARM 1: Cohort 1: ipsilateral RT Cohort 2: ipsilateral TORS + neck dissection +/- CRT ARM 2: bilateral RT +/- CT	Field reduction (RT limited to neck at risk)	Adverse events	N/A
NCT03729518 The Abramson Cancer Center, Penn Medicine Est. 12/2025	Active Not Recruiting	Phase 2 (n= est. 150)	P16+ OPSCC, T0-T3 N0-N2b <5 positive lymph nodes (AJCC 7th ed.)	TORS + RT (50 Gy) to tumor bed + RT (45 Gy) to contralateral neck +/- CT	Target volume reduction (RT to tumor bed and contralateral neck only)	LRCR	N/A

*Transoral robotic surgery (TORS) may be complemented by adjuvant neck dissection when clinically indicated.

PI, principal investigator, number; AJCC, American Joint Committee on Cancer; ed, edition; LRCR, locoregional control rate; LCR, local control rate; CUP, cancer of unknown primary; PSRT, parapharyngeal sparing Radiotherapy; UNRT, Unilateral neck radiotherapy; MDADI, MD Anderson Dysphagia Inventory; DSS, disease specific survival; PFS, progression free survival; LRCR, locoregional control rate; TOS, transoral surgery; IMRT, Intensity Modulated Radiation Therapy; Fx, fractions; OS, overall survival; DFS, disease free survival; TLM, transoral Laser Microsurgery; EATOS, endoscopic assisted transoral surgery; PORT, postoperative radiotherapy; N/A, not applicable; SCCUP, squamous cell carcinoma of unknown primary; HNSCC, Head and neck squamous cell carcinoma.

##### Interventional and randomized clinical trials

Several prospective interventional trials have evaluated postoperative RT dose reduction following TORS in selected patient populations. The Mayo Clinic MC1273 “DART – **D**e-escalated **A**djuvant **R**adio**T**herapy”) trial (NCT01932697) included 80 patients with p16-positive stage III–IV OPSCC (AJCC 7th edition) who underwent TORS with negative surgical margins (24). Patients were stratified by the presence of extranodal extension (ENE). Cohort A (intermediate risk, no ENE; n=37) received 30 Gy in 1.5-Gy fractions twice daily over two weeks with concurrent weekly docetaxel (15 mg/m²). Cohort B (high risk, ENE present; n=43) received the same regimen plus a simultaneous integrated boost to the involved nodal levels with ENE to 36 Gy in 1.8-Gy fractions twice daily. At a median follow-up of 35.7 months, reported outcomes included a 2-year locoregional control rate of 96.2% (cohort A: 100%; cohort B: 93.0%), progression-free survival of 91.1%, distant metastasis-free survival of 94.9%, and overall survival of 98.7%. Grade ≥3 toxicity rates were 2.5% at pre-RT and 0% at one and two years post-RT, with only one patient requiring temporary feeding tube placement (REF). These encouraging phase 2 results formed the basis for the subsequent phase III DART-HPV trial (MC1675), discussed below.

Similarly, the SIRS trial (SInai Robotic Surgery: NCT02072148) evaluated risk-adapted adjuvant therapy following TORS in patients with early-stage p16-positive OPSCC. Patients were stratified into low-, intermediate-, and high-risk groups, receiving no adjuvant RT, 50 Gy adjuvant RT, or 56 Gy with weekly cisplatin, respectively. All risk groups demonstrated excellent progression-free survival rates (25).

The ECOG 3311 trial (Eastern COoperative Group: NCT01898494) enrolled 495 patients with p16-positive OPSCC and stratified them into four treatment arms based on pathological risk factors. Low-risk patients (pT1-2, N0-1, negative margins, no ENE) underwent observation; intermediate-risk patients (pT3, or N2a-N2b with ≤1 mm ENE, or positive margins without ENE) received either low-dose RT (50 Gy) or standard-dose RT (60 Gy), and high-risk patients (positive margins with ENE, or gross ENE) received intensity-modulated RT (66 Gy in 33 fractions) with weekly cisplatin (40 mg/m²). Two-year PFS rates were 96.9%, 94.9%, 96.0%, and 90.7% for groups A, B, C, and D, respectively (26).

The AVOID trial (Alternative Volumes of Oropharyngeal Irradiation for De-intensification: NCT02159703) was a prospective clinical study investigating omission of RT to the resected primary tumor bed following TORS in HPV-positive OPSCC. At a median follow-up of 2.4 years, a single patient recurred at the primary site, yielding a 2-year local control rate of 98.3% and a 2-year local recurrence-free survival of 97.9% (95% CI, 86.1%–99.7%). One patient (1.7%) developed a regional neck recurrence and two (3.3%) developed distant metastases. Overall survival was 100% at the time of analysis. Favorable toxicity profiles were reported (27).

The FIND trial (NCT03281499) evaluated 22 patients with p16-positive head and neck carcinoma of unknown primary who underwent diagnostic or therapeutic TORS. Based on pathological findings, patients in whom a primary tumor was identified received pharyngeal-sparing RT limited to the involved mucosal volume, while patients without an identifiable primary tumor received standard extended mucosal RT. Pharyngeal-sparing RT demonstrated excellent oncologic outcomes and superior swallowing-related quality of life, with MDADI scores approximately 20 points higher at 24 months compared with non–pharyngeal-sparing approaches (28).

The phase III DART-HPV (De-escalated Adjuvant Radio Therapy in HPV-positive OPSCC) trial (Mayo Clinic MC1675; NCT02908477), published in *The Lancet Oncology* (2025), enrolled 228 patients with intermediate- or high-risk HPV-associated OPSCC (AJCC 7th edition, stage III–IV), of whom 194 patients proceeded to protocol treatment and primary analysis (130 DART, 64 SOC), randomized in a 2:1 ratio. Patients received either de-escalated adjuvant CRT (30 Gy in 1.5 Gy fractions twice daily over 2 weeks, with a simultaneous boost to 36 Gy for patients with extranodal extension [ENE], combined with docetaxel 15 mg/m²) or standard adjuvant treatment (60 Gy in 2 Gy fractions once daily over 6 weeks, with weekly cisplatin 40 mg/m²). At a median follow-up of 37.3 months, the cumulative chronic grade 3 or higher toxicity rate at 3 to 24 months was significantly lower in the DART arm compared with the standard arm (3% vs 11%; p=0.042), as was the PEG tube dependence rate (2% vs 8%; p=0.039).

Subgroup analyses revealed an important differential effect: in the intermediate-risk group (defined as lymphovascular invasion, perineural invasion, >2 positive lymph nodes, lymph node >3 cm, or pT3 primary tumor), 2-year PFS was 98% with DART versus 95% with SOC, suggesting a potential benefit of de-escalation in this subgroup. In contrast, among ENE-positive patients, 2-year PFS was 81% with DART versus 97% with SOC, confirming that de-escalation is not appropriate for this high-risk subgroup. A secondary analysis further demonstrated that postoperative circulating tumor HPV DNA (ctHPVDNA) was significantly associated with recurrence risk, suggesting that ctHPVDNA assessment may improve patient selection for treatment de-escalation (25, 25b). It should be noted, however, that the trial was not formally powered to assess non-inferiority; the observed hazard ratios for PFS (HR 4.76) and OS (HR 1.68) make non-inferiority of the DART regimen highly unlikely. These results therefore support de-escalation in carefully selected intermediate-risk patients, while warranting caution against broader adoption—particularly in ENE-positive disease (29).

##### Ongoing and observational studies

Several ongoing trials are further investigating adjuvant treatment de-escalation following TORS. The PATHOS trial (NCT02215265) aims to enroll 1,269 patients with HPV-positive OPSCC undergoing primary surgery followed by risk-adapted adjuvant therapy. Primary outcome measures include swallowing function assessed by MDADI scores and water swallow test, quality of life, toxicity, disease-free survival, recurrence, and distant metastasis (10).

Two additional studies focus on omission of RT to the primary tumor site. The SUPERIOR trial (NCT06578871), which has not yet started recruitment, will randomize patients with p16-positive carcinoma of unknown primary to surgery with standard adjuvant therapy versus surgery without intensity-modulated RT or with RT limited to the ipsilateral neck. Trial NCT06554158 is a single-arm study in which all patients receive 50 Gy RT to the neck at risk following TORS, with omission of RT to the primary tumor site.

Finally, the LONE-RANGR2 study (NCT06167291) aims to reduce adjuvant RT by limiting radiation fields to the neck at risk. In an estimated cohort of 240 patients with p16-positive HNSCC, safety outcomes are assessed based on adverse event incidence.

#### De-escalation based on HPV-DNA

Recent evidence suggests a correlation between circulating cell-free HPV DNA (cfHPVDNA) dynamics and treatment response or recurrence in HPV-associated oropharyngeal squamous cell carcinoma (OPSCC), raising interest in cfHPVDNA as a biomarker for risk stratification and guidance of adjuvant therapy de-escalation (30). Currently, five clinical trials are investigating the role of cfHPVDNA in informing treatment de-intensification strategies. Summary can be found in **Table 4**.

**Table 4:** Clinical trials evaluating HPVDNA-guided de-escalation strategies following TORS.

Study number, first author or PI, date of publication	Study status	Study type (sample size)	Patient inclusion	Intervention	Role of cfHPVDNA	De-escalated treatment component	Primary outcome	Key findings
NCT05419089 (SIRS 2.0) PI: Chai, R. Est. 06/2027	Recruiting	Phase 2 (n= est. 83)	P16+ T1N0-2B, T2N0-2B OPSCC (AJCC 7th ed.), cfHPVDNA +	TORS * with negative postoperative cfHPVDNA + Low risk: observation ** High risk: CRT (46 Gy + cisplatin) ***	Risk stratification for adjuvant therapy selection	Observation for cfHPVDNA-negative low-risk patients; reduced-dose CRT (46 Gy + cisplatin) for high-risk patients	LRR	N/A
NCT05714657 PI: Lin, A. Est. 04/2026	Recruiting	Phase 2 (n= est. 104)	P16+/HPV+ OPSCC, T0-T3 N0-N1 (AJCC 8th ed.)	TORS* with negative postoperative cfHPVDNA + reduced dose RT (30 Gy)	Eligibility criterion for adjuvant treatment de-escalation	Reduced-dose adjuvant radiotherapy (30 Gy)	LRCR	N/A
NCT05387915 PI: Bates, J. E. Est. 05/2026	Recruiting	Phase 2 (n= est. 33)	P16+/HPV+ OPSCC, pT1-2, pN0-1, cM0 (AJCC 8th ed.), cfHPVDNA +	TORS + Postoperative - cfHPVDNA: reduced dose RT (3weeks 15 fx) Postoperative + cfHPVDNA: standard RT	Risk stratification for postoperative radiotherapy	Reduced-dose adjuvant radiotherapy for cfHPVDNA-negative patients; standard-dose radiotherapy for cfHPVDNA-positive patients	MDADI	N/A
NCT06088381 (SAVAL) PI: Molitoris, J.K. Est. 10/2027	Recruiting	Phase 2 (n= est. 61)	P16+ OPSCC/SCCUP, T0-3, N0-N1, and M0 disease (AJCC 8th ed.), cfHPVDNA +	TORS + Low risk/intermediate risk **** + negative ctDNA: observation PET/CT and ctDNA observation High risk + positive ctDNA: standard of care therapy	Eligibility criterion for omission of adjuvant therapy	Observation only for cfHPVDNA-negative low- or intermediate-risk patients	PFS	N/A
NCT06445114 PI: Zumsteg, Z.S. Est. 03/2032	Not Yet Recruiting	Phase 2 (n= est. 50)	P16+ T0-3N0–2 OPSCC/SCC/CUP (AJCC 8th ed.)	TORS + weekly 40mg/m2 cisplatin + ***** Low Risk + HPV DNA negativity: 30 Gy Low Risk + HPV DNA positivity: 40 Gy High Risk + HPV DNA negativity: 40 Gy High Risk + HPV DNA positivity: 50 Gy	Combined biomarker and pathological risk stratification	Risk-adapted reduced-dose adjuvant radiotherapy (30–50 Gy based on HPV DNA status and risk group)	PFS	N/A
NCT04572100 Rosenberg A. Oct 2023 (ABSTRACT ESMO)^28^	Completed	Phase 1 (n= 46)	HPV+/p16+ OPSCC, N1 (>=3cm), N2-N3 or T3-T4 (AJCC 8th ed.)	Induction therapy + Low risk > 50% reduction: TORS/RT Low risk <50% and >30% reduction or high risk >50% reduction: CT + low dose RT	Surveillance biomarker for treatment response and recurrence detection	Not applicable (biomarker validation study)	Feasibility of serial HPV-DNA blood sample collection, Correlation between circulating HPV-DNA levels and response to chemotherapy	At baseline, cfHPV-DNA was detectable in 98% of patients, with 72% achieving HPV-DNA clearance after two cycles. Clearance was associated with a 100% one-year progression-free survival (PFS), while persistence correlated with a 79% one-year PFS. cfHPV-DNA demonstrated 100% sensitivity in detecting recurrences, with a positive predictive value of 80%. Induction chemotherapy yielded high response rates, with 91% of patients experiencing ≥30% tumor shrinkage and 73% achieving ≥50% shrinkage.

* Transoral robotic surgery (TORS) may be complemented by adjuvant neck dissection when clinically indicated.

** <4 positive lymph nodes, ≤2 mm extra nodal extension, and perineural invasion or lymphovascular invasion alone

*** 4 positive lymph nodes, gross extra nodal extension, positive margins, N2c disease and/or the combination of both perineural invasion and lymphovascular invasion

**** TORS pT1–3 N1–2 with negative surgical margins/no or ≤1mm microscopic extranodal extension

***** depending on risk stratification patients will receive an adjuvant boost of RT (10/20 Gy)

PI, principal investigator; n, number; AJCC, American Joint Committee on Cancer; ed, edition; cfHPVDNA, Circulating cell-free HPV DNA; LRR, locoregional recurrence; N/A, not applicable; LRCR, locoregional control rate; MDADI, MD Anderson Dysphagia Inventory; SCCUP, squamous cell carcinoma of unknown primary; PFS, progression free survival; HNC, head and neck cancer; cTTMV HPVDNA, circulation tumor tissue modified viral HPV DNA;.

##### Completed and reporting studies

The NCT04572100 trial, completed in 2023, evaluated cfHPVDNA as a biomarker for treatment response and recurrence in conjunction with tumor response to induction chemotherapy. Although the final peer-reviewed publication is pending, results presented in an ESMO 2023 abstract included 46 evaluable patients and demonstrated a sensitivity of 100% and a positive predictive value of 80% for recurrence detection (31).

##### Interventional trials guiding adjuvant therapy based on cfHPVDNA

The SIRS 2.0 trial (NCT05419089) aims to enroll 199 patients with undetectable cfHPVDNA following TORS. Patients are stratified into a low-risk OPSCC group managed with observation and a high-risk OPSCC group receiving de-intensified adjuvant therapy consisting of 46 Gy radiotherapy with weekly cisplatin. CfHPVDNA levels are monitored every three months for two years, with the primary endpoint being two-year locoregional control rate (LRCR).

Similarly, the single-arm phase II trial NCT05714657 evaluates reduced-dose adjuvant radiotherapy (30 Gy) in post-TORS patients with cfHPVDNA negativity. Outcome measures include locoregional control, progression-free survival, metastasis-free survival, overall survival, dysphagia, and quality of life. Results are anticipated in April 2026.

The NCT05387915 trial, which is currently recruiting, plans to include 33 patients undergoing TORS for low-stage OPSCC. Patients are stratified based on postoperative cfHPVDNA status: those with undetectable cfHPVDNA after surgery receive reduced-dose radiotherapy, whereas those with detectable postoperative cfHPVDNA are treated with standard-dose radiotherapy. Functional outcomes, recurrence rates, and survival outcomes will be compared with matched controls from the ECOG 3311 trial. Study completion is expected in May 2026.

Trial NCT06445114 stratifies patients based on postoperative cfHPVDNA status and pathological risk factors, assigning reduced radiotherapy doses accordingly. Results from this study are expected by March 2032.

##### Observational and exploratory studies

The SAVAL study (NCT06088381) is an observational single-arm trial enrolling 61 patients with p16-positive squamous cell carcinoma following TORS. Patients are stratified according to pathological risk factors and cfHPVDNA status. Low- and intermediate-risk patients with undetectable cfHPVDNA undergo observation for two years, whereas high-risk patients receive adjuvant therapy. The study explores whether adjuvant radiotherapy may be omitted in intermediate-risk patients with cleared cfHPVDNA. Results are expected in October 2027.

#### Induction therapy

Another de-escalation strategy within TORS-based treatment paradigms involves intensification of neoadjuvant therapy to stratify patients for omission, minimization, or de-intensification of adjuvant therapies. Completed and ongoing clinical trials evaluating induction strategies include chemotherapy, immunotherapy, vaccination-based approaches, local anesthetics, and viral vector therapies. An overview of these studies is provided in **Table 5**.

**Table 5:** Clinical trials on induction therapy followed by TORS.

Study number, first author or PI, date of publication	Study status	Study type (sample size)	Patients characteristics	Intervention	Purpose of induction therapy	Primary outcome	Key findings
NCT03618134 Ma T.M. Oct 2023 (32)	Completed	Phase 1b/2 (n= 19)	HPV+/p16+ OPSCC, T0–3 N0-N2b (max 2 nodes)	Arm 1: SBRT (25Gy) + durvalumab +TORS * Arm 2: SBRT (25 Gy)+ durvalumab + tremelimumab + TORS *	Exploratory biological modulation (immune priming combined with local radiotherapy)	Adverse events, PFS	Neoadjuvant SBRT associated with immunotherapy before TORS is not effective nor safe and should not be considered as a de-escalation strategy.
NCT03107182 (OPTIMA 2) Rosenberg A.J. Jun 2024 (30)	Completed	Phase 2 (n= 73)	HPV+/p16+ OPSCC, N2-N3 or T3-T4 or (AJCC 7^th^ ed.)	Induction therapy (nivolumab + nab-paclitaxel + carboplatin) + Low risk: RT (50 Gy) or TORS Intermediate risk: TFHX + cisplatin High risk: regular dose CRT (70–75 Gy)	Response-adapted risk stratification for adjuvant de-escalation	Tumor shrinkage	Induction immunochemotherapy followed by response-adapted locoregional therapy, showed excellent oncological and functional outcomes, although it did not distinguish itself in DRR. Patients undergoing robotic surgery showed a 67% complete pathologic response, a 100% survival rate, and a complete omission of RT.
NCT01612351Weiss M. May 2018 (28, 29)	Active Not Recruiting	Phase 2 (n= 40)	Stage III/IV HNSCCA (AJCC 7^th^ ed.)	Induction therapy (carboplatin + paclitaxel + lapatinib) + TOS* pN0 or pN1 + negative margins: observation R1 (microscopic +/or <5mm margins) or extracapsular extension or pN2a/pN2b or perineural invasion or lymphovascular space invasion): ipsilateral chemoradiation + cisplatin (weekly) pN2c/pN3or R2 resection: bilateral concurrent chemoradiation + cisplatin	Response-adapted risk stratification to enable RT omission	ORR	Neoadjuvant immunochemotherapy followed by surgery and risk adapted adjuvant therapy is feasible. It showed high response rates, acceptable functional outcomes and a high omission rate of radiotherapy (77%).
NCT05108870 PI: Rosenberg, A. Est. 01/2026	Recruiting	Phase 1 Phase 2 (n= est. 98)	HPV+/p16+ OPSCC N1≥ 3, N2-N3, T3-T4	Phase 1: dose finding Phase 2: HB-201 + HB-202 + CT + TORS/RT+ CRT	Immune priming and tumor downstaging using viral vector–based vaccination	Dose, DRR	N/A
NCT06747390 PI: Carey, R. Recruiting Est. 11/2028	Recruiting	Phase 1 (n= est. 30)	T1-T4, N0-N3 OPSCC/HNSCC	lidocaine 1% into primary tumor bed + TORS* vs TORS*	Local biological modulation prior to surgery	Adverse events, pTR	N/A
NCT04277858 (NECTORS) Study chair: Sadegh, N. Est. 08/2026	Recruiting	Phase 2 (n= est. 60)	P16+ OPSCC, T1-T3, N0-N2, M0 (AJCC 7th ed.)	Neoadjuvant docetaxel + cisplatin/carboplatin + TORS *	Tumor downstaging and response assessment prior to surgery	PFS	N/A
NCT04988074 (MINIMA) PI: Fakhry, C. Est. 08/2026	Recruiting	Phase 2 (n= est. 32)	HPV+/p16+, Stage II -III, stage I with N1/N2, no T4 (AJCC 8th ed.)	neoadjuvant Cemiplimab +/- carboplatin/paclitaxel De-escalation group: TORS/RT (42 Gy) Non/minimally de-escalation group: surgery + RT/CRT (60 Gy) **	Response-adapted de-escalation following immunotherapy	PFS, MDADI, SSQ,	N/A
NCT02760667 Siegel R.S. Est. 12/2023 (ABSTRACT ASCO) (31)	Active Not Recruiting	Phase 2 (n= 20)	OPSCC, T1N1-N2, T2N1-N2, T3N0-N2, early T4, M0 (AJCC 7th ed.)	Induction: Cisplatin + Docetaxel + TORS/TLM	Tumor downstaging to reduce need for adjuvant radiotherapy	DSS	Cisplatin + docetaxel followed by TORS and neck dissection could be an effective treatment for OPSCC, while omitting adverse events of RT. 18 patients were alive at mean follow up of 40.7 months and had no evidence of disease. Three patients experienced recurrence within 2.2 months and 2 patients died due to metastatic disease.

*Transoral robotic surgery (TORS) may be complemented by adjuvant neck dissection when clinically indicated.

**all groups receive postoperative cemiplimab for 4 weeks.

PI, principal investigator; n, number; AJCC, American Joint Committee on Cancer; ed, edition; SBRT, stereotactic body radiotherapy; PFS, progression free survival; DRR, ≥50% tumor shrinkage by RECIST 1.1; HNSCC, Head and neck squamous cell carcinoma; TOS, transoral surgery; ORR, Overall response rate; N/A, not applicable; pTR, pathologic tumor response; MDADI, MD Anderson Dysphagia Inventory; SSQ, Sydney Swallow Questionnaire; TLM, transoral Laser Microsurgery; DSS, disease specific survival.

##### Completed and reporting studies

Weiss et al. evaluated induction chemoimmunotherapy followed by transoral surgery and risk-stratified adjuvant therapy in patients with high-grade HNSCC. Patients were stratified based on pretreatment clinical evaluation. Reported outcomes demonstrated excellent functional results, with omission of adjuvant radiotherapy achieved in 77% of patients (32, 33).

The OPTIMA 2 trial (Rosenberg et al., 2024) included 77 patients with stage III–IV HPV-positive OPSCC (AJCC 8^th^ edition) treated with induction immunochemotherapy consisting of nivolumab, nab-paclitaxel, and carboplatin. Based on response to induction therapy, patients were stratified into three treatment arms: low-risk patients treated with TORS or radiotherapy, intermediate-risk patients receiving de-escalated chemoradiotherapy, and high-risk patients receiving standard-dose chemoradiotherapy. Reported results demonstrated excellent overall survival. Improved functional outcomes were observed specifically in patients managed with de-escalated chemoradiotherapy compared with those receiving standard-dose chemoradiotherapy; outcomes in patients treated with TORS were not separately reported (34).

Trial NCT02760667, completed in 2023, investigated the functional and oncologic outcomes of induction chemotherapy with cisplatin and docetaxel in 20 patients with advanced OPSCC. Results presented in an ASCO 2020 abstract suggested that induction therapy followed by TORS was effective while mitigating radiotherapy-related adverse effects; final peer-reviewed results remain unpublished (35).

Trial NCT03618134 evaluated induction immunotherapy combined with stereotactic body radiotherapy (SBRT) followed by TORS in 19 patients with locally advanced HPV-positive HNSCC. High recurrence rates were observed, leading investigators to conclude that this treatment strategy was unsuitable for clinical implementation (36).

##### Ongoing clinical trials

The MINIMA trial (NCT04988074), currently recruiting, aims to enroll 32 patients undergoing induction therapy with cemiplimab. Patients are stratified into a de-escalated treatment group and a non- or minimally de-escalated treatment group. The de-escalated group receives either TORS or low-dose radiotherapy, whereas the non- or minimally de-escalated group undergoes surgery followed by postoperative radiotherapy or chemoradiotherapy. Study completion is anticipated in August 2026.

The NECTORS trial (NCT04277858) is an ongoing study investigating induction chemotherapy followed by TORS, with primary outcomes including progression-free survival, disease-specific survival, overall survival, and quality of life. Results are expected in August 2026.

A phase I/II clinical trial conducted by the University of Chicago (NCT05108870) is evaluating TheraT^®^ Vector–based vaccination prior to TORS or risk-adapted chemoradiotherapy. This study investigates the safety and efficacy of HB-201 and HB-202 vaccines administered in combination with chemotherapy as induction therapy, with the hypothesis that this approach may enhance tumor shrinkage. An estimated 98 patients with advanced stage HPV-positive OPSCC will be enrolled, with study completion anticipated in January 2026.

Finally, the phase I trial NCT06747390 is evaluating intratumoral injection of lidocaine 1% prior to TORS in patients with HPV-positive and HPV-negative HNSCC. Emerging evidence suggests that lidocaine may exert direct antitumor effects beyond its known anesthetic properties. Preclinical studies suggest tumor cell proliferation inhibition and apoptosis induction through activation of intracellular signaling pathways, including calcium-mediated mitochondrial dysfunction and caspase activation (37, 38). In addition, lidocaine may suppress prometastatic pathways by blocking voltage-gated sodium channels involved in tumor cell migration and invasion (39). Clinical data further support this concept, as peri-tumoral lidocaine administration has been associated with improved oncologic outcomes in breast cancer patients (39). Participants included in the abovementioned study are allocated to injection and non-injection arms prior to surgery. Primary outcome measures include adverse events and pathological tumor response, with results expected later in 2026.

## Discussion

### Optimization of TORS techniques

Qualitative data on optimization strategies for TORS in OPSCC remain limited. In this review, only eight registered clinical trials addressed perioperative or technical optimization. Most were characterized by small sample sizes, limiting statistical power and generalizability. Consequently, firm conclusions regarding optimal perioperative strategies remain difficult to draw. The role of corticosteroids as part of perioperative analgesic regimens remains inconclusive. Clayburgh et al. reported modest improvements in short-term pain scores, dietary tolerance, and median length of hospital stay following perioperative dexamethasone administration, whereas Larsen et al. in a smaller randomized cohort (be it with a lower dose of corticosteroids even in their high-dose arm), were unable to replicate these findings (12, 13). Earlier work by Scott et al. suggested a partial reduction in short-term postoperative pain with corticosteroid use (19), highlighting persistent heterogeneity in reported outcomes and study designs.

Technical optimization of robotic platforms has been explored in several studies, although the available evidence is predominantly derived from single-center experiences, pilot studies with small sample sizes, and non-randomized designs. The introduction of the SP robotic system has been shown to be safe, feasible, and effective for OPSCC (13). These findings are supported by institutional experience from the same center, demonstrating comparable oncologic and functional outcomes between the SP system and the previously used Si platform (40). Comparative analyses by Costantino et al. further corroborated these findings by demonstrating equivalence between the SP and da Vinci Xi systems (41). Notably, the SP system may offer practical advantages in terms of docking efficiency and reduced console time (40, 41). In contrast, our own institutional experience did not demonstrate clinically meaningful advantages of the Xi platform over the Si system (42).

While results from ongoing trials are awaited, future research should focus on identifying patient- and procedure-specific advantages of the SP system, which has recently been introduced into the European market. Additional areas of interest include the development of effective multimodal pain management strategies and improved tumor visualization techniques, such as fluorescence-guided surgery, to further enhance surgical precision and safety.

### TORS as an alternative treatment to (chemo)radiotherapy

Several clinical trials have demonstrated that TORS achieves short- and long-term oncologic outcomes comparable to radiotherapy in early-stage OPSCC (14–16, 19–23). However, only one published trial specifically focused on a p16-positive population (20), underscoring a relative paucity of HPV-stratified randomized data. While both RT and TORS are associated with distinct toxicity profiles, comparative functional outcomes remain nuanced.

Scott et al. observed significant improvements in objective swallowing function between one and three years after treatment in both RT and TORS cohorts, with a suggested advantage for TORS. However, these improvements did not translate into clinically meaningful differences in patient-reported quality of life (19). Recent meta-analyses have confirmed oncologic and functional equivalence between TORS-based and non-surgical approaches, supporting current American Society of Clinical Oncology (ASCO) guideline recommendations (8, 43, 44). According to these guidelines, TORS is an appropriate treatment option for T1–T2 OPSCC or recurrent disease, provided that adequate transoral exposure is achievable, the tumor is lateralized, an R0 resection is likely, and extranodal extension is absent (**Figure 1**).

Importantly, most ongoing comparative trials incorporate HPV stratification and increasingly compare TORS not only with RT but also with other surgical modalities, such as transoral laser microsurgery, which may further refine treatment selection in the near future.

### TORS as de-escalation therapy

Accumulating evidence - although predominantly derived from phase 2 trials with relatively limited sample sizes, potential selection bis, and relatively short follow-up- suggests that reduction of adjuvant RT in selected low- and intermediate-risk HPV-positive OPSCC patients may preserve oncologic outcomes while improving functional results (24–29). The recently published phase III DART-HPV trial provides the first randomized data in this setting: the 2-year PFS of 92.3% met the prespecified noninferiority benchmark, and chronic grade ≥3 toxicity was significantly reduced (3% vs 11%; p=0.042) (29). However, the trial was not powered for non-inferiority, and the observed hazard ratios raise caution regarding disease control, particularly in high-risk subgroups. As such, de-escalation of adjuvant RT cannot yet be considered definitively proven safe and should be approached carefully, with rigorous patient selection excluding pN2 disease and ENE-positive cases. Two principal de-escalation strategies have emerged: reduction of RT dose and omission of RT to selected resected tumor targets. Direct comparisons between de-escalation protocols remain challenging due to heterogeneity in surgical margin definitions, surgical techniques, pathological risk stratification, and adjuvant treatment regimens. These inconsistencies limit cross-trial comparability and highlight the need for standardized definitions and reporting frameworks. The growing body of evidence supports a trend toward personalized, risk-adapted treatment strategies that aim to reduce toxicity while maintaining oncologic control, although mature phase 3 data confirming equivalence remain limited. Future research should aim to refine patient selection criteria, harmonize de-escalation protocols, and identify optimal combinations of surgical and adjuvant strategies to facilitate broader clinical implementation.

### Biomarker driven de-escalation

Despite increasing interest, evidence supporting biomarker-driven de-escalation strategies remains limited. The NCT04572100 trial demonstrated a correlation between circulating cell-free HPV DNA (cfHPVDNA) clearance and favorable progression-free survival, as well as high sensitivity for recurrence detection. These findings have been corroborated by two recent systematic reviews (11, 30). Notably, a secondary analysis of the phase III DART-HPV trial demonstrated that postoperative ctHPVDNA detection was significantly associated with disease progression: patients with detectable ctHPVDNA after surgery had an 18-month PFS of only 73.1%, compared with 95.8% for patients without detectable ctHPVDNA (p<0.01). At three months post-treatment, the prognostic value was even more pronounced, with PFS rates of 25% versus 97.2% for ctHPVDNA-positive and ctHPVDNA-negative patients, respectively (p<0.0001) (25b). These findings provide supportive evidence from a randomized trial that ctHPVDNA may hold potential as a biomarker to inform adjuvant therapy de-escalation decisions (29).

Beyond OPSCC, emerging data in HPV-driven cervical carcinoma further support the role of cell-free HPV DNA as a post-treatment surveillance tool (45, 46).

Collectively, these findings suggest that cfHPVDNA may facilitate more personalized postoperative management and potentially inform revised standardized surveillance protocols. However, large-scale prospective trials are required to determine whether post-treatment cfHPVDNA levels can reliably serve as a stratification tool for guiding adjuvant therapy decisions and de-escalation strategies in routine clinical practice.

### Induction therapy

Several phase 2 studies have demonstrated that induction immunochemotherapy followed by TORS in patients with advanced stage HNSCC can achieve favorable oncologic outcomes while enabling omission of adjuvant RT in a subset of patients (32–34). Weiss et al. reported omission of adjuvant RT in 77% of patients (32, 33), whereas in the OPTIMA 2 trial, de-escalated treatment was applied only to patients classified as low-risk based on response to induction therapy (34). It should be noted, however, that in many cases, patients ultimately received triple-modality treatment (induction systemic therapy, surgery, and adjuvant radiotherapy), which raises the question of whether this approach truly constitutes de-escalation or rather represents treatment intensification. Furthermore, level 1 evidence from randomized phase 3 trials is currently lacking. These response-adapted strategies highlight the potential of induction therapy to guide personalized treatment pathways, but their role as a genuine de-escalation strategy requires further scrutiny. In contrast, neoadjuvant stereotactic body RT combined with immunotherapy followed by TORS was associated with unacceptable recurrence rates and cannot be considered a safe or viable treatment approach (36). These negative findings reflect the limitations not of induction therapy per se, but of the subsequent de-escalation strategy applied in this context. In this regard, the KEYNOTE-689 trial demonstrated improved event-free survival with perioperative pembrolizumab in operable locally advanced head and neck squamous cell carcinoma, including oropharyngeal carcinoma - with postoperative treatment being less intensive in responders. However, oropharyngeal and oral cavity carcinomas were pooled in the subgroup analysis, limiting the interpretation of TORS-specific implications (47). Future research should therefore focus on optimizing patient selection for de-escalation following induction therapy, rather than questioning the concept of induction therapy itself.

## Conclusion

In this scoping review, we provide a comprehensive overview of clinical trials evaluating transoral robotic surgery in OPSCC and identify key gaps in the existing literature. As evidence continues to accumulate, TORS appears to achieve oncologic and functional outcomes comparable to non-surgical approaches while offering unique opportunities for treatment de-intensification in carefully selected patient populations. Central to this evolution is robust risk stratification, in which emerging biomarkers such as cfHPVDNA may play a crucial future role. Ongoing advances in robotic technology, optimization of perioperative management, and refinement of de-escalation and induction strategies are essential to further improve patient outcomes. Continued prospective, HPV-stratified, and biomarker-integrated trials will be critical to defining the optimal role of TORS within contemporary multidisciplinary management of OPSCC.

## Data Availability

The original contributions presented in the study are included in the article/supplementary material, further inquiries can be directed to the corresponding author/s.
